# Co-Occurrence Patterns of Pathological High-Risk Factors and Predictive Value of PET Metabolic Features in Lung Adenocarcinoma: A Retrospective Cohort Study of 1292 Cases

**DOI:** 10.3390/cancers18213435

**Published:** 2026-10-23

**Authors:** Qiuyu Leng, Yu Huang, Bowen Zhao, Zuhan Geng, Chi Zhang, Yongde Liao

**Affiliations:** Department of Thoracic Surgery, Union Hospital, Tongji Medical College, Huazhong University of Science and Technology, Wuhan 430022, China; mitic1996@163.com (Q.L.);

**Keywords:** lung adenocarcinoma, pathological high-risk factors, co-occurrence, positron emission tomography, maximum standardized uptake value, spread through air spaces, lymphovascular invasion

## Abstract

**Background/Objectives:** Co-occurrence patterns among pathological high-risk factors in lung adenocarcinoma remain poorly characterized, and whether preoperative ^18^F-fluorodeoxyglucose (^18^F-FDG) positron emission tomography/computed tomography (PET/CT) predicts them is unsettled. We mapped their interrelationships and evaluated the predictive value of preoperative maximum standardized uptake value (SUVmax). **Methods:** We retrospectively enrolled 1292 consecutive patients with resected invasive lung adenocarcinoma (May 2020–September 2023) and extracted six high-risk factors: spread through air spaces (STAS), visceral pleural invasion (VPI), lymphovascular invasion (LVI), occult lymph node metastasis (OLNM), and solid and micropapillary components. Pairwise co-occurrence was quantified by the Phi coefficient with Bonferroni correction; logistic regression and receiver operating characteristic (ROC) analysis assessed the predictive value of SUVmax. High-risk burden was defined as ≥3 concurrent factors. **Results:** STAS (29.8%) and the micropapillary component (24.4%) were the most prevalent. All 15 pairwise associations were positive and significant after Bonferroni correction; the strongest enrichment was between LVI and OLNM (observed-to-expected [O/E] ratio 5.85, Phi = 0.41, *p* < 0.001), with no mutual exclusivity. SUVmax independently predicted all six factors (e.g., solid component, odds ratio [OR] 1.283; STAS, OR 1.225; micropapillary component, OR 1.141; all *p* < 0.001). For high-risk burden, SUVmax achieved an area under the curve (AUC) of 0.838, which was numerically higher than that for any individual factor. At a cut-off of 3.0, sensitivity was 86.8% and the negative predictive value was 96.8% (bootstrap 95% confidence interval [CI] 0.807–0.865). **Conclusions:** High-risk pathological factors co-occur rather than segregate. Preoperative SUVmax is independently associated with all six factors but is best positioned to identify high-risk burden rather than individual features, although a clinical benefit remains to be demonstrated.

## Simple Summary

Lung adenocarcinoma is the most common type of lung cancer. After surgery, pathologists can identify certain features that signal a higher risk of the cancer returning, but knowing about them beforehand would help doctors plan treatment. In this study, we examined 1292 patients who had lung cancer surgery. We looked at how six of these high-risk features relate to one another, and whether a value taken from routine PET/CT scans before surgery, called SUVmax, can predict them. We found that these features tend to occur together rather than to exclude one another, and that SUVmax was associated with all six. More importantly, SUVmax was better at spotting patients who had several high-risk features than any single one. These results suggest PET/CT may help doctors identify the most aggressive tumors, although further studies are needed before these changes are used in practice.

## 1. Introduction

Lung adenocarcinoma, the most prevalent histological subtype of non-small cell lung cancer (NSCLC), exhibits marked heterogeneity in biological behavior and clinical outcome. The 2021 WHO Classification of Thoracic Tumours partitions invasive nonmucinous lung adenocarcinoma into five predominant histological patterns: lepidic, acinar, papillary, micropapillary, and solid. Among these, micropapillary and solid predominant subtypes confer the poorest prognosis [1,2]. Accordingly, the 2021 WHO classification designates both patterns as high-grade histological features [1]. Beyond these architectural subtypes, postoperative pathological examination frequently identifies additional invasive characteristics strongly associated with adverse outcomes, including spread through air spaces (STAS), visceral pleural invasion (VPI), lymphovascular invasion (LVI), and occult lymph node metastasis (OLNM) [3,4,5,6]. The presence of these factors directly informs adjuvant chemotherapy decisions, radiation therapy indications, and the intensity of postoperative surveillance, as reflected in the National Comprehensive Cancer Network (NCCN) guidelines [7]. Clinical risk factors such as smoking history and larger tumor size further modify this risk.

The prognostic significance of individual high-risk factors has been firmly established. Kadota et al. reported that among patients with small (≤2 cm) stage I lung adenocarcinomas treated with limited resection, STAS-positive tumors were associated with a 5-year cumulative recurrence incidence of 42.6%, compared with 10.9% for STAS-negative tumors (*p* < 0.001) [5]. This was independently corroborated by Warth et al., who found that extensive intra-alveolar tumor spread significantly compromised both overall and disease-free survival in a cohort of 569 patients [8]. Building on this evidence, the IASLC Pathology Committee formalized a three-tier grading system for invasive pulmonary adenocarcinoma that demonstrated independent prognostic value for both recurrence-free and overall survival across multiple international cohorts, with interobserver reproducibility of κ = 0.617 [9]: grade 1 (lepidic predominant), grade 2 (acinar or papillary predominant with < 20% high-grade patterns), and grade 3 (any tumor with ≥20% solid, micropapillary, or complex glandular patterns). The architectural classification itself has also been validated as a stage-independent predictor of survival [10]. Despite this wealth of prognostic data, the interrelationships and co-occurrence patterns among these factors remain poorly mapped. Each factor has largely been studied in isolation: prior studies have demonstrated that VPI independently predicts survival and pleural recurrence [4], that lymphatic permeation and vascular invasion each represent independent prognostic factors in stage I adenocarcinoma [11], and that OLNM is most prevalent in solid and micropapillary predominant subtypes [6]. Yet, these investigations have not systematically examined how the factors associate with one another within the same tumor. Whether these factors co-occur more often than expected from their individual frequencies remains unclear; such co-occurrence would indicate shared invasive biology. Co-occurrence less often than expected would instead indicate alternative routes of spread. Neither direction has been quantified [12]. For example, it remains unknown whether STAS and VPI, two major routes of dissemination through airspaces and pleural surfaces respectively, are more or less likely to occur together. Similarly, the relationship between the solid and micropapillary histological patterns and lymphovascular and occult nodal involvement has not been quantified. Elucidating these co-occurrence patterns could reveal fundamental invasive mechanisms and inform risk-stratification models that account for factor clustering rather than simple summation.

The diagnostic workup of a suspicious pulmonary nodule integrates contrast-enhanced CT, ^18^F-fluorodeoxyglucose positron emission tomography/computed tomography (^18^F-FDG PET/CT), and tissue sampling by endobronchial ultrasound-guided transbronchial needle aspiration (EBUS-TBNA) or surgery. Preoperative ^18^F-FDG PET/CT is routinely recommended for mediastinal lymph node staging in patients with NSCLC, with the maximum standardized uptake value (SUVmax) serving as a semiquantitative biomarker of tumor metabolic activity [13]. Elevated SUVmax has been linked to high-risk pathological subtypes, including solid component and lymph node metastasis, in clinical stage IA lung adenocarcinoma [14]. Among resected stage I patients, solid predominant histology, characterized by elevated FDG avidity, was identified as an independent predictor of early extrathoracic multisite recurrence [15]. The predictive value of SUVmax for individual high-risk factors, however, is inconsistent. A recent radiomics study reported that SUVmax alone yielded only 74.7% accuracy for identifying aggressive histological patterns such as solid and micropapillary, substantially lower than a combined PET/CT texture-based radiomics model, suggesting inherent limitations of SUVmax in capturing the full spectrum of high-risk histology [16]. Furthermore, the association between SUVmax and pathological aggressiveness, though reported for tumor size, solid component, and lymph node metastasis [14,15], remains unsettled for specific factors, particularly the micropapillary component and STAS [16]. Some investigators have proposed that SUVmax functions primarily as a nonspecific marker of overall tumor biological aggressiveness rather than a predictor of individual histological patterns: it correlates with tumor volume doubling time [17], and nomograms integrating SUVmax with CT-derived tumor characteristics outperform conventional T staging in predicting survival [18]. These discrepancies highlight the need for large-scale, comprehensive analyses that evaluate SUVmax against multiple high-risk factors simultaneously while controlling for confounding clinical variables.

To address these gaps, we conducted a retrospective cohort study of 1292 consecutive patients with resected invasive lung adenocarcinoma who underwent preoperative PET/CT and had complete pathological assessments, with three specific objectives. First, we systematically characterized the prevalence and pairwise co-occurrence patterns of six pathological high-risk factors (STAS, VPI, LVI, OLNM, solid component, and micropapillary component) using Phi correlation coefficients and observed-to-expected ratios. Second, we evaluated the independent predictive value of preoperative SUVmax for each factor using multivariable logistic regression with adjustment for age, sex, tumor size, and location. Third, we assessed the discriminative performance of SUVmax not only for individual factors but also for high-risk burden, defined as the concurrent presence of three or more factors. We tested two hypotheses. First, the six high-risk factors would exhibit non-random co-occurrence, with positive associations between biologically related features. Second, SUVmax would predict high-risk burden better than any individual factor.

## 2. Materials and Methods

### 2.1. Study Design and Participants

**Figure 1:**
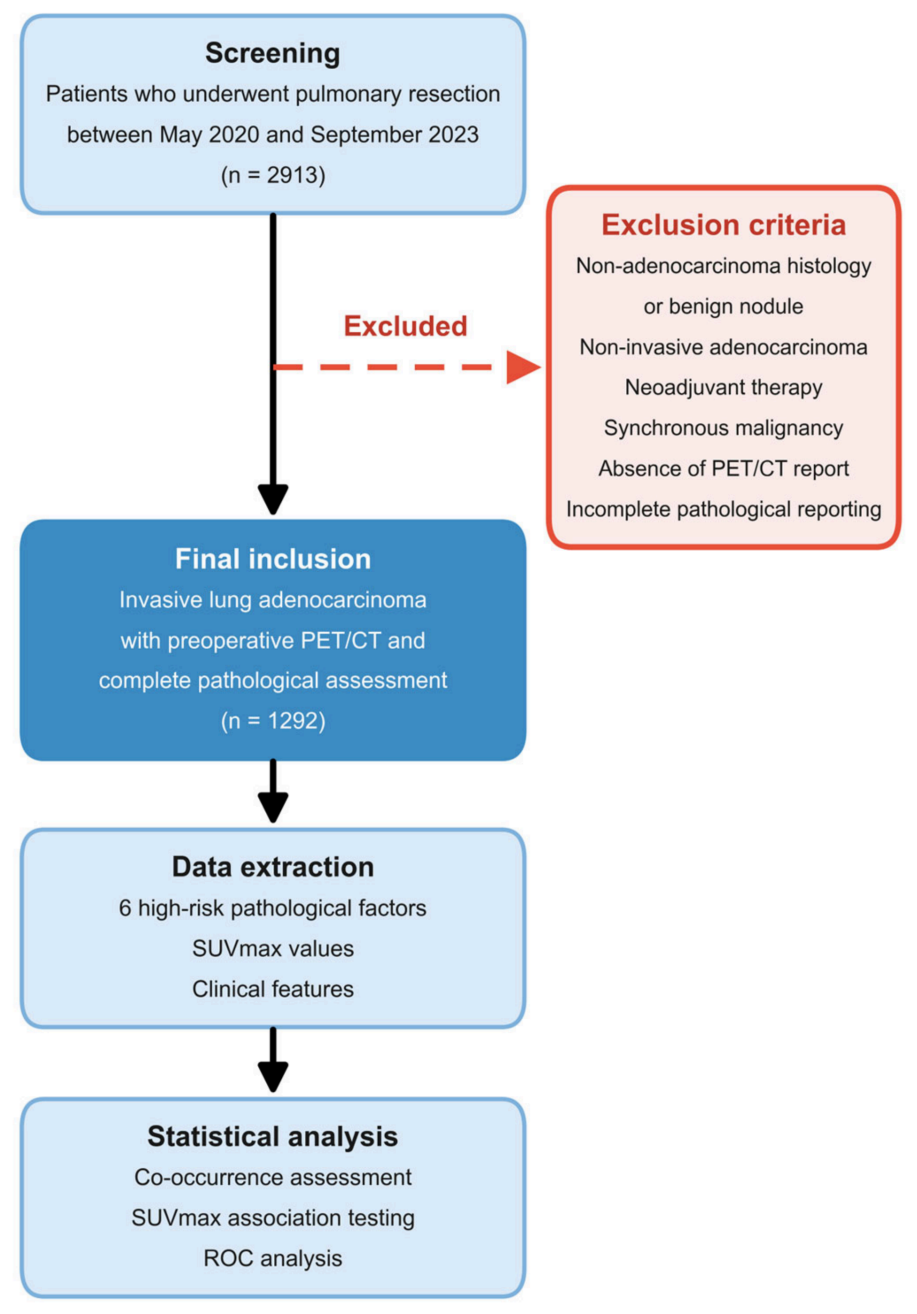
Study flowchart. Of 2913 patients who underwent pulmonary resection between May 2020 and September 2023, 1621 were excluded according to predefined criteria (non-adenocarcinoma histology or benign nodule, non-invasive adenocarcinoma, neoadjuvant therapy, synchronous malignancy, absence of PET/CT report, or incomplete pathological reporting). A total of 1292 patients with invasive lung adenocarcinoma were included; tumors negative for all six high-risk factors were retained in the analysis. Statistical analyses comprised assessment of co-occurrence among the six high-risk factors, univariable testing of the association between SUVmax and each factor, and receiver operating characteristic analysis.

We retrospectively reviewed consecutive patients who underwent surgical resection (lobectomy, segmentectomy, or wedge resection with lymph node dissection or sampling) for lung adenocarcinoma at Wuhan Union Hospital between May 2020 and September 2023. Patients were included if they met all of the following criteria: (1) pathologically confirmed invasive lung adenocarcinoma; (2) preoperative ^18^F-FDG PET/CT performed within one month before surgery; (3) complete postoperative pathological reporting of the six high-risk factors; and (4) availability of clinical data including age, sex, nodule location, and maximum diameter. Exclusion criteria comprised neoadjuvant chemotherapy, radiotherapy, or targeted therapy before surgery; synchronous or metachronous other malignancies; benign or non-adenocarcinomatous nodules; non-invasive adenocarcinoma, including adenocarcinoma in situ and minimally invasive adenocarcinoma; and absence of a PET/CT report or complete pathological reporting (Figure 1). The final eligible cohort comprised 1292 patients with invasive lung adenocarcinoma. Tumors negative for all six high-risk factors were retained in the primary analysis, so that prevalence and co-occurrence were estimated in the full eligible population. Ethical approval was obtained from the Institutional Ethics Committee of Wuhan Union Hospital (protocol code UHCT250609, approved on 22 June 2025), and the requirement for informed consent was waived due to the retrospective nature of the study.

### 2.2. Variable Definitions

Six pathological high-risk factors were retrospectively extracted by two independent investigators from institutional postoperative pathology reports issued by board-certified pathologists, with disagreements resolved through consensus discussion.

Spread through air spaces (STAS) was defined as the presence of tumor cell clusters within alveolar spaces beyond the main tumor margin on hematoxylin-eosin staining. Visceral pleural invasion (VPI) was recorded when tumor cells invaded beyond the elastic layer of the visceral pleura (PL1 or PL2) or penetrated the pleural surface (PL3). Lymphovascular invasion (LVI) was defined as the presence of tumor cells within lymphatic or vascular spaces. Occult lymph node metastasis (OLNM) was defined as unexpected lymph node metastasis detected on postoperative pathology that was not suspected preoperatively based on imaging or intraoperative findings, including peribronchial, N1, and N2 station involvement.

Solid component was defined as any solid growth pattern recorded in the pathology report (>0%). A subgroup analysis within solid-component-positive tumours, using the IASLC ≥20% threshold, was additionally performed. Micropapillary component was defined as a micropapillary growth pattern proportion ≥5% recorded in the pathology report. Because the minimum recorded percentage of micropapillary components in our institutional pathology reports was 5%, the ≥5% threshold was operationally equivalent to any presence (>0%) in this cohort. Continuous percentage data were available for 246 of the 251 solid-component-positive and 314 of the 315 micropapillary-positive tumors; in these patients, solid and micropapillary components were additionally treated as continuous variables (percentage) to assess dose–response relationships.

### 2.3. PET/CT Acquisition and SUVmax Measurement

All patients fasted for at least 6 h before the examination, and imaging was performed only after blood glucose had been confirmed to be within the normal range (≤11.1 mmol/L). ^18^F-FDG was administered intravenously at 3.8–5.5 MBq/kg (0.10–0.15 mCi/kg), and image acquisition began 50–60 min after injection. PET/CT was performed on an integrated scanner (GE Discovery VCT, GE Healthcare, Milwaukee, WI, USA). The same scanner and acquisition parameters were used throughout the study period. A low-dose CT scan was acquired from the head to the pelvis, and PET data were acquired in three-dimensional mode at 2 min per bed position over 6–8 bed positions. After scatter and decay correction, images were reconstructed with attenuation correction and reviewed in axial, sagittal, and coronal planes on a dedicated workstation. The maximum standardized uptake value (SUVmax) of the primary lung nodule was retrieved from the preoperative PET/CT reports reviewed by board-certified nuclear medicine physicians. SUV was normalized to body weight (SUVbw), the most widely used convention in oncological FDG PET/CT. SUVmax was treated as a continuous variable in all analyses.

### 2.4. Covariates

Age (years), sex (male/female), nodule location (right upper/middle/lower lobe; left upper/lower lobe), and maximum nodule diameter (mm) were collected from electronic medical records. Nodule location was further dichotomized as upper lobe versus non-upper lobe for subgroup analyses. Nodule size was dichotomized at the median value (<22 mm vs. ≥22 mm).

### 2.5. Statistical Analysis

Continuous variables were expressed as median (interquartile range, IQR) after confirmation of non-normal distribution using the Shapiro–Wilk test. Categorical variables were expressed as frequency (percentage) with 95% confidence intervals (CI) calculated using the Clopper–Pearson exact method. Comparisons between groups were performed using the Mann–Whitney U test for two groups or the Kruskal–Wallis test with Dunn’s post-hoc test for multiple groups. Categorical variables were compared using the chi-square test or Fisher’s exact test, as appropriate. Temporal trends in the annual detection rates of the six factors were assessed by linear regression across study years.

### 2.6. Co-Occurrence Analysis

Pairwise co-occurrence frequencies were calculated and presented as a 6 × 6 matrix. The observed-to-expected (O/E) ratio was computed for each pair under the assumption of independence. The Phi coefficient was used to quantify the strength of association between binary high-risk factors. Fisher’s exact test was applied for all pairwise comparisons, with Bonferroni correction for multiple comparisons (15 pairs, adjusted significance threshold α = 0.0033). A network graph was constructed to visualize positive and negative associations, with node size proportional to factor prevalence, edge width proportional to the absolute Phi coefficient, and edge color indicating positive (red) or negative (blue) correlation.

### 2.7. SUVmax Association Analysis

The relationship between SUVmax and each binary high-risk factor was assessed using the Mann–Whitney U test. Spearman’s rank correlation coefficient (ρ) was used to evaluate associations between SUVmax and continuous percentages of solid and micropapillary components. The relationship between SUVmax and the ordinal number of concurrent high-risk factors was evaluated using the Kruskal–Wallis test.

### 2.8. Multivariable Analysis

Binary logistic regression was performed for each high-risk factor as the dependent variable, with SUVmax, age, sex, nodule size, and nodule location as independent variables. For each model, the reference group comprised patients negative for that specific factor, including those negative for all six factors. The threshold of three or more concurrent factors was derived from the present dataset rather than specified a priori and is therefore treated as exploratory. It was retained because the discriminative performance of SUVmax was nearly identical for thresholds of three (area under the curve [AUC] 0.838) and four (AUC 0.839) or more factors, whereas only 76 patients (5.9%) harbored four or more factors. Because the six factors differ in their biological meaning and in the threshold used to define positivity, the burden count was treated as a pragmatic aggregate index. For the analysis of high-risk burden, patients with three or more concurrent factors were compared with those carrying fewer than three factors. Odds ratios (ORs) with 95% CI were reported. Model fit was assessed using the Akaike information criterion (AIC) and pseudo-R^2^ (McFadden’s R^2^).

Model discrimination and cut-off stability were assessed using 1000 bootstrap resamples. In each resample, the optimal SUVmax cut-off was re-derived by the Youden index; optimism-corrected sensitivity and specificity, together with 95% confidence intervals for AUC and cut-off, were reported.

### 2.9. Predictive Performance

Receiver operating characteristic (ROC) curves were constructed to evaluate the discriminative ability of SUVmax for each high-risk factor and for high-risk burden, defined as the concurrent presence of ≥3 high-risk factors. The AUC, with 95% confidence intervals estimated using the method of DeLong, optimal cut-off determined by the Youden index, sensitivity, and specificity were calculated.

### 2.10. Subgroup and Sensitivity Analyses

To assess the robustness of our findings, we repeated all analyses using solid component ≥20% as the threshold. We additionally assessed whether temporal changes in pathological reporting influenced the principal findings: the multivariable model for high-risk burden was repeated with year of surgery added as a covariate, and the pairwise Phi coefficients were recomputed within each year of surgery. All statistical analyses were performed using Python (version 3.13) with SciPy (1.12), statsmodels (0.14), and scikit-learn (1.4). A two-sided *p*-value < 0.05 was considered statistically significant, except for pairwise co-occurrence analyses where Bonferroni-corrected *p* < 0.0033 was applied. Figures were generated using Matplotlib 3.8 and Seaborn 0.13.

## 3. Results

### 3.1. Patient Characteristics and Prevalence of High-Risk Factors

Of the 2913 patients who underwent pulmonary resection between May 2020 and September 2023, 1621 were excluded (non-adenocarcinoma histology or benign nodule, non-invasive adenocarcinoma, neoadjuvant therapy, synchronous malignancy, absence of PET/CT report, or incomplete pathological reporting). The final cohort comprised 1292 patients with invasive lung adenocarcinoma (Figure 1); tumors negative for all six high-risk factors were retained in the analysis. The median age was 60 years (interquartile range [IQR] 54–66), and 544 (42.1%) were male. The median preoperative SUVmax was 2.2 (IQR 1.1–5.0), and the median nodule diameter was 20 mm (IQR 15–25 mm). The distribution of concurrent high-risk factors was as follows: 0 factors in 635 patients (49.1%), 1 factor in 260 (20.1%), 2 factors in 207 (16.0%), 3 factors in 114 (8.8%), 4 factors in 52 (4.0%), 5 factors in 20 (1.5%), and 6 factors in 4 (0.3%). Baseline characteristics stratified by the number of concurrent high-risk factors are presented in Table 1.

**Figure 2:**
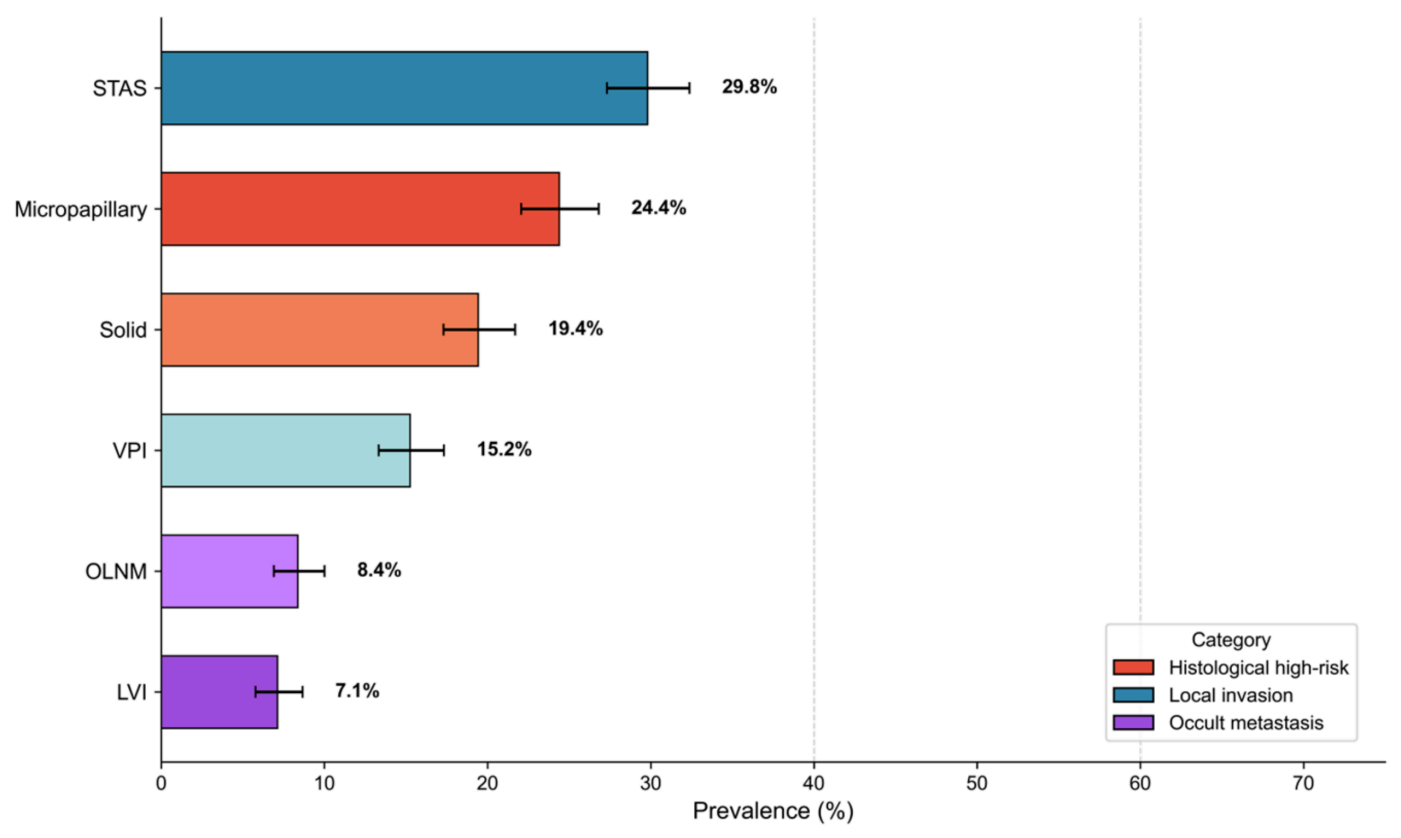
Prevalence of pathological high-risk factors in the study cohort (n = 1292). Data were derived from postoperative pathology reports for resections performed between 2020 and 2023. Factors are grouped into three categories: histological high-risk features, comprising the solid and micropapillary components; local invasion features, comprising STAS and VPI; and occult metastasis, comprising LVI and OLNM. Error bars represent 95% confidence intervals calculated using the Clopper–Pearson exact method.

The prevalence of each high-risk factor is summarized in Table 2 and illustrated in Figure 2. STAS was the most common factor, present in 385 patients (29.8%, 95% confidence interval [CI] 27.3–32.4%), followed by micropapillary component in 315 (24.4%, 95% CI 22.1–26.8%) and solid component in 251 (19.4%, 95% CI 17.3–21.7%). VPI, OLNM, and LVI were observed in 197 (15.2%, 95% CI 13.3–17.3%), 108 (8.4%, 95% CI 6.9–10.0%), and 92 (7.1%, 95% CI 5.8–8.7%) patients, respectively.

### 3.2. Co-Occurrence Patterns and Inter-Factor Associations

**Figure 3:**
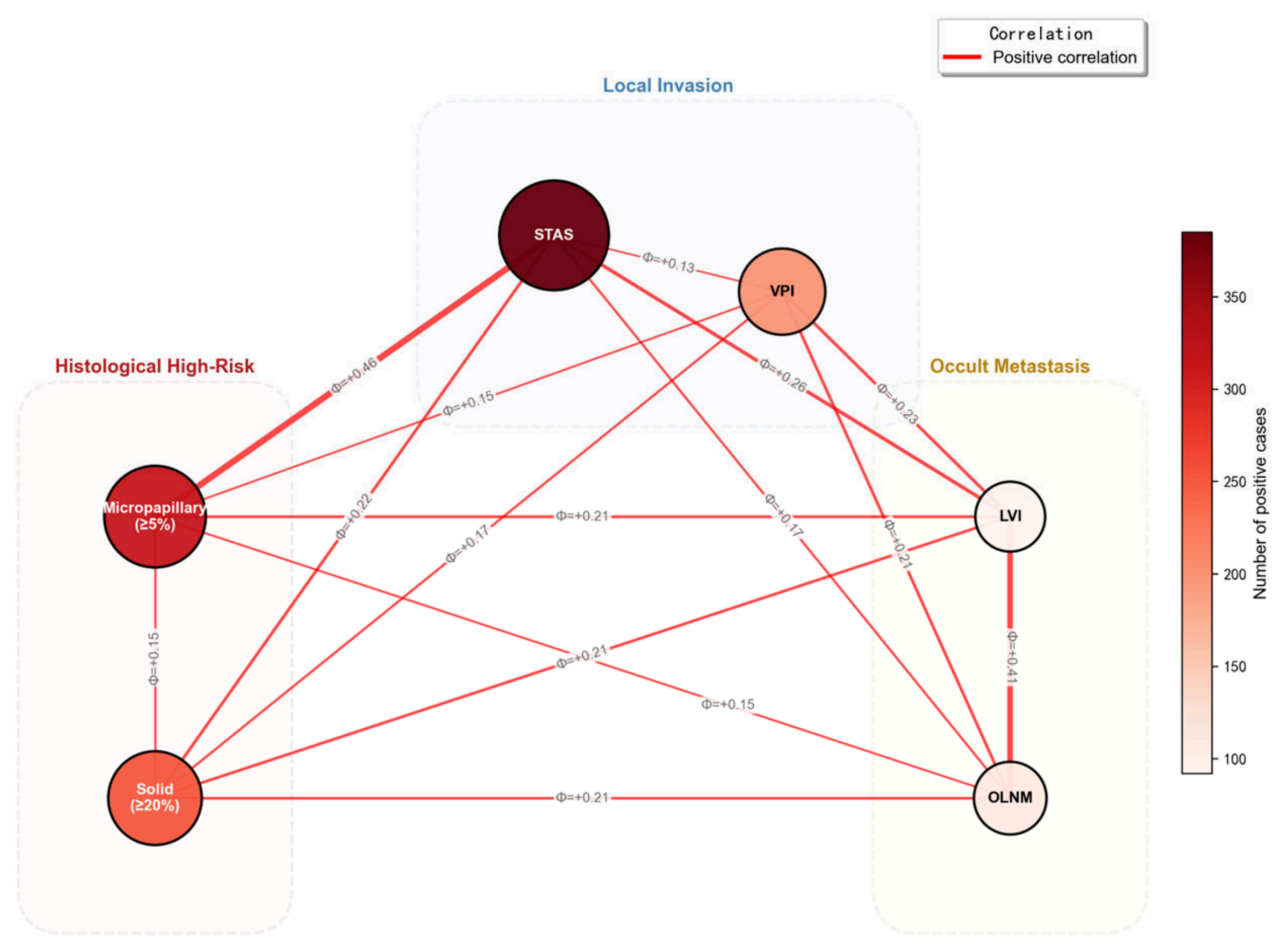
Co-occurrence network of pathological high-risk factors (n = 1292). Node size and colour intensity are proportional to the number of positive cases for each factor. Edges are shown for every pair that remained significant after Bonferroni correction (*p* < 0.0033); all 15 pairs met this criterion, and every edge represents a positive association, with width proportional to the absolute Phi coefficient and labels giving the Phi value. Factors are grouped into histological high-risk features, local invasion features, and occult metastasis.

**Figure 4:**
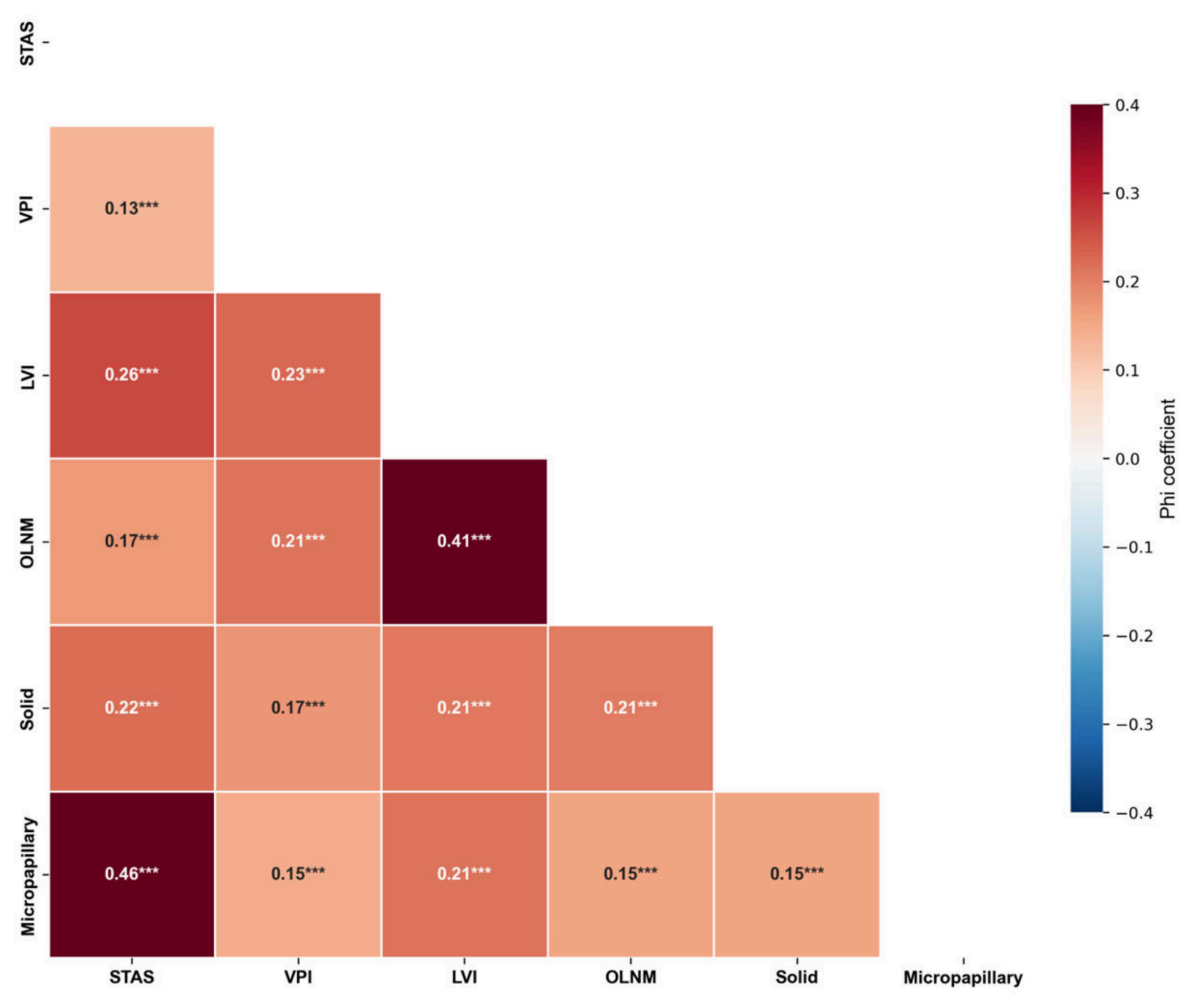
Phi correlation matrix of pathological high-risk factors. The lower triangle displays pairwise Phi coefficients with significance levels (*** *p* < 0.001). The Bonferroni-corrected significance threshold for 15 pairwise comparisons was α = 0.0033. Red shading indicates positive correlation (co-occurrence) and blue shading indicates negative correlation (mutual exclusivity); all 15 pairs showed positive coefficients, so no blue cell appears in the matrix. Phi coefficients were interpreted as negligible (<0.10), weak to moderate (0.10–0.30), and moderate to strong (≥0.30). The color intensity reflects the magnitude of the Phi coefficient.

Pairwise co-occurrence analysis revealed positive associations between all 15 pairs of the six high-risk factors, and every association remained significant after Bonferroni correction (Figure 3 and Figure 4, and Table 3). The strongest enrichment was observed between LVI and OLNM (observed-to-expected [O/E] ratio 5.85; Phi coefficient = 0.41; *p* < 0.001), indicating that these two features co-occurred nearly six times more often than expected under independence. LVI and OLNM also contributed to three further associations with an O/E ratio above 2.6 (VPI–LVI, O/E = 2.92; VPI–OLNM, O/E = 2.67), whereas the largest Phi coefficient of the entire matrix was found between STAS and micropapillary component (Phi = 0.46; O/E = 2.25; *p* < 0.001). The remaining pairs showed more moderate enrichment, with O/E ratios ranging from 1.48 to 2.52 (Table 3).

No pair of factors showed mutual exclusivity. Across the 15 pairs, the O/E ratio ranged from 1.48 (STAS and VPI) to 5.85 (LVI and OLNM). All six features were therefore more likely to occur together than to segregate. LVI and OLNM contributed to the four most enriched associations, placing them at the center of the network. STAS and the micropapillary component formed the pair with the largest correlation coefficient.

### 3.3. Association Between SUVmax and High-Risk Factors

#### 3.3.1. Univariate Analysis

In univariate analysis, patients with a solid component had significantly higher SUVmax than those without (median 5.9 vs. 1.9, Mann–Whitney U test *p* < 0.001). SUVmax was likewise elevated in patients with VPI (5.3 vs. 1.9, *p* < 0.001), LVI (6.5 vs. 2.1, *p* < 0.001), OLNM (6.5 vs. 2.0, *p* < 0.001), STAS (4.5 vs. 1.7, *p* < 0.001), and micropapillary component (4.2 vs. 1.9, *p* < 0.001) compared with their respective negative groups.

**Figure 5:**
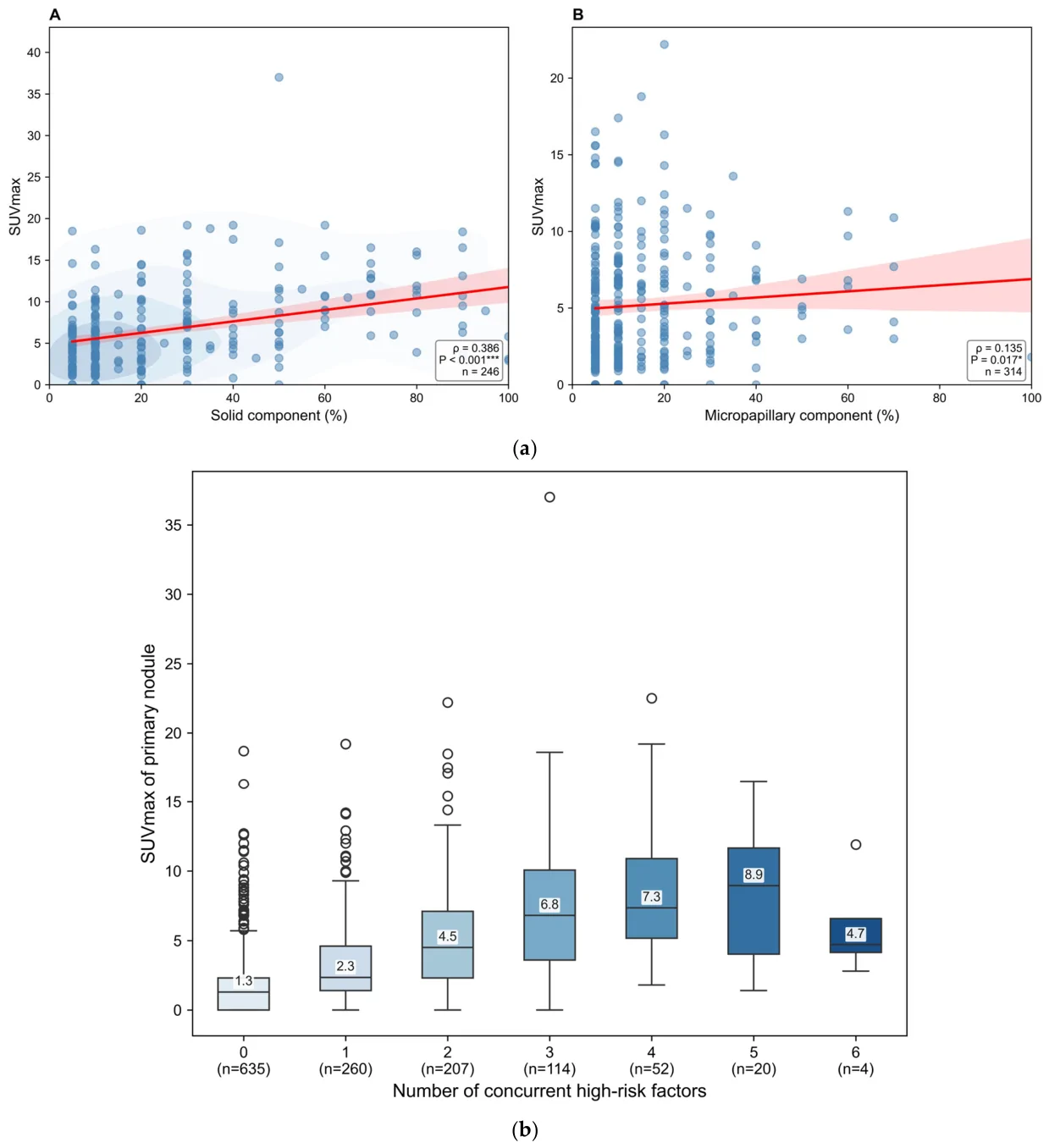
(**a**). Association between SUVmax and continuous percentages of pathological high-risk factors. Scatter plots with linear regression lines (red) and 95% confidence bands (pink) showing Spearman correlations between SUVmax and (**A**) solid component percentage (ρ = 0.386, *p* < 0.001, n = 246) and (**B**) micropapillary component percentage (ρ = 0.135, *p* = 0.017, n = 314). (**b**). Dose–response relationship between SUVmax and the number of concurrent high-risk factors. Box plots show the distribution of SUVmax stratified by the number of concurrent high-risk factors (Kruskal–Wallis H = 426.0, *p* < 0.001). Boxes represent the interquartile range (IQR) with the median value labeled above the median line within each box; whiskers extend to 1.5 × IQR; open circles indicate outliers. Sample sizes are shown below each group. * *p* < 0.05; *** *p* < 0.001.

Spearman correlation analysis showed a moderate positive correlation between SUVmax and the continuous percentage of solid component (ρ = 0.386, *p* < 0.001, n = 246), whereas only a weak correlation was observed between SUVmax and micropapillary percentage (ρ = 0.135, *p* = 0.017, n = 314) (Figure 5a). Detailed univariate comparisons are presented in Table 4.

A clear dose–response relationship was observed between the number of concurrent high-risk factors and SUVmax (Kruskal–Wallis H = 426.0, *p* < 0.001; Figure 5b). The median SUVmax increased with the number of concurrent high-risk factors, from 1.3 (IQR 0.0–2.3) in patients with no high-risk factor to 2.3 (IQR 1.4–4.6) with 1 factor, 4.5 (IQR 2.3–7.1) with 2 factors, 6.8 (IQR 3.6–10.1) with 3 factors, 7.3 (IQR 5.2–10.9) with 4 factors, and 8.9 (IQR 4.0–11.7) with 5 factors; in the four patients carrying all six factors the median was 4.7 (IQR 4.1–6.6).

#### 3.3.2. Multivariable Logistic Regression

After adjusting for age, sex, nodule size, and location, SUVmax remained an independent predictor of all six high-risk factors (Table 5). The strongest association was observed for the solid component (adjusted odds ratio [aOR] 1.283, 95% CI 1.229–1.339, *p* < 0.001). The remaining five factors showed a narrow gradient, from STAS (aOR 1.225) and LVI (1.223) through OLNM (1.188) and VPI (1.178) to the micropapillary component (1.141); all *p* < 0.001. Male sex was independently associated with solid component (aOR 1.994, 95% CI 1.464–2.716, *p* < 0.001), whereas nodule size was associated with VPI (aOR 1.034, 95% CI 1.009–1.059, *p* = 0.007).

In the subgroup analysis using a ≥20% threshold for solid component (n = 246), the predictive effect of SUVmax was even stronger (aOR 1.272, 95% CI 1.172–1.381, *p* < 0.001), with a pseudo-R^2^ of 0.152 compared with 0.166 in the primary analysis.

#### 3.3.3. Predictive Performance (ROC Analysis)

**Figure 6:**
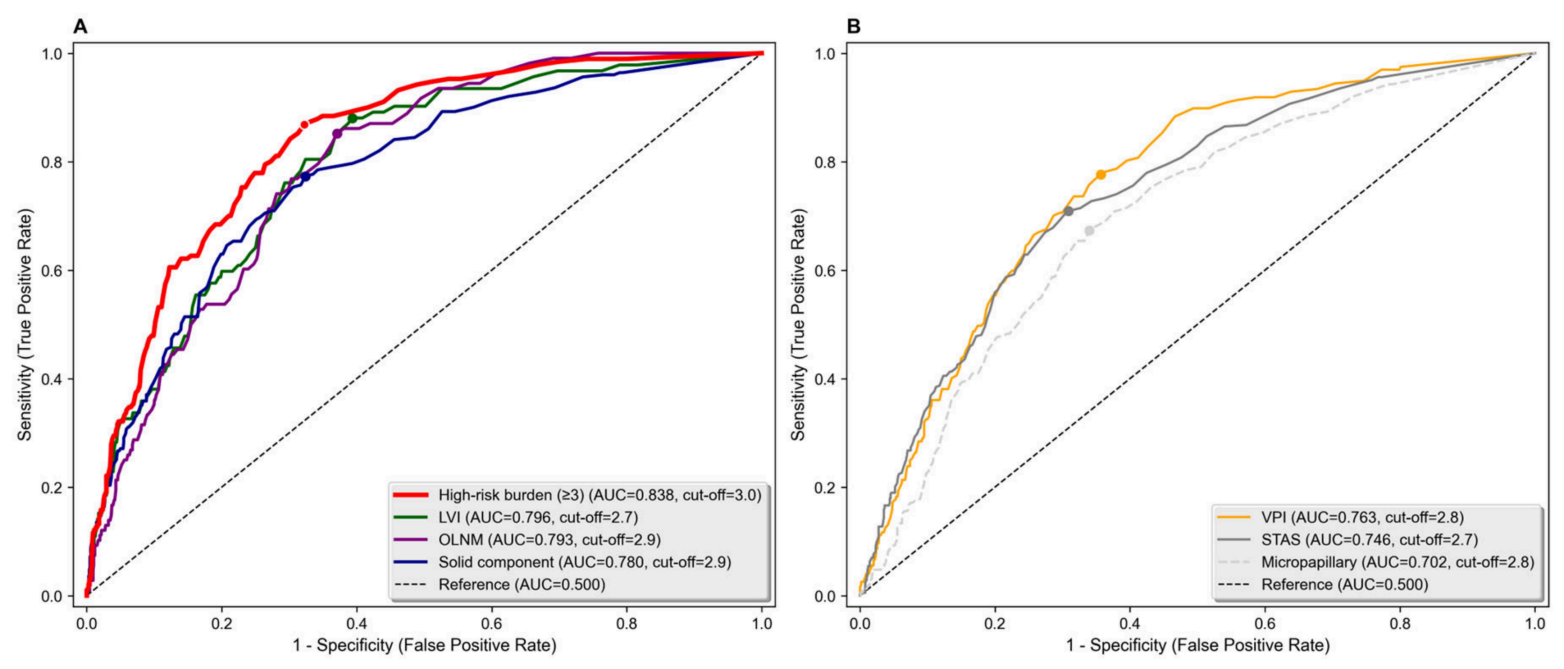
Receiver operating characteristic (ROC) curves evaluating the discriminative performance of SUVmax. (**A**) High-risk burden (≥3 concurrent factors) and the three factors with the highest individual AUCs. (**B**) The remaining three factors. The dashed diagonal line represents the reference classifier (AUC = 0.500). AUC values and optimal cut-offs (determined by the Youden index) are shown in the figure. High-risk burden showed the highest discriminative performance.

The discriminative performance of SUVmax for individual high-risk factors was moderate (AUC 0.702–0.796; Table 6 and Figure 6). The highest AUCs were observed for LVI (0.796, cut-off 2.7, sensitivity 88.0%, specificity 60.7%) and OLNM (0.793, cut-off 2.9, sensitivity 85.2%, specificity 62.9%), followed by solid component (0.780, cut-off 2.9, sensitivity 77.3%, specificity 67.6%) and VPI (0.763, cut-off 2.8, sensitivity 77.7%, specificity 64.3%). STAS (0.746, cut-off 2.7, sensitivity 70.9%, specificity 69.1%) and micropapillary component (0.702, cut-off 2.8, sensitivity 67.3%, specificity 66.0%) showed the lowest, yet still significant, discrimination.

SUVmax showed higher discriminative performance for high-risk burden (≥3 concurrent factors, n = 190) than for any individual factor, with an AUC of 0.838 (cut-off 3.0, sensitivity 86.8%, specificity 67.8%). At this cut-off the positive predictive value was 31.7% and the negative predictive value was 96.8%, reflecting the low prevalence of high-risk burden (14.7%) in the full cohort. The subgroup analysis for solid component ≥20% yielded an AUC of 0.699 (cut-off 6.7, sensitivity 58.3%, specificity 76.3%). This analysis was performed within the 246 solid-component-positive patients with recorded percentages, so its denominator differs from that of the primary analysis. Bootstrap internal validation (1000 resamples) confirmed the discrimination for high-risk burden (AUC 0.838, 95% CI 0.807–0.865; optimism-corrected sensitivity 0.856, specificity 0.676), whereas the optimal cut-off was less stable (3.0, 95% CI 2.7–4.1) (Table S3).

### 3.4. Clinical and Temporal Associations

Nodule size was significantly associated with the presence of all six high-risk factors: STAS (37.3% in ≥22 mm vs. 24.8% in < 22 mm), VPI (21.8% vs. 10.9%), LVI (10.6% vs. 4.8%), OLNM (12.2% vs. 5.8%), solid component (26.1% vs. 15.0%), and micropapillary component (29.9% vs. 20.7%) (all *p* ≤ 0.0002) (Table S2). No significant difference was found between upper-lobe and non-upper-lobe locations for any high-risk factor (all *p* > 0.05) (Table S1).

Temporal analysis from 2020 to 2023 revealed a borderline increasing trend in the STAS detection rate (5.5% to 44.2%, nominal linear regression *p* = 0.047). This trend did not, however, survive correction for the six factors examined (Holm-adjusted *p* = 0.28); the trends for LVI and the micropapillary component were of similar magnitude but likewise non-significant (*p* = 0.074 and *p* = 0.067). No factor showed a significant temporal trend after correction. Repeating the multivariable model for high-risk burden with year of surgery added as a covariate did not materially alter discrimination (AUC 0.834 vs. 0.838), and the pairwise Phi coefficients remained positive within each year of surgery, indicating that the co-occurrence structure was not explained by temporal changes in pathological reporting.

## 4. Discussion

In this retrospective cohort of 1292 patients with resected invasive lung adenocarcinoma, we systematically investigated the co-occurrence patterns of six pathological high-risk factors and evaluated the independent predictive value of preoperative SUVmax. Our findings revealed significant non-random associations among these factors: every pairwise association was positive, the strongest enrichment linked lymphovascular invasion to nodal involvement, and the largest correlation coefficient linked spread through air spaces to the micropapillary component.

SUVmax was an independent predictor of all six high-risk factors, most strongly for the solid component, STAS, and LVI, and least strongly for the micropapillary component. Importantly, SUVmax achieved its best discriminative performance not for any individual pathological feature, but for high-risk burden, defined as the concurrent presence of three or more factors.

While prior studies have examined these factors in isolation [4,6,10,11], few have attempted to characterize their pairwise interrelationships or to reposition SUVmax from a single-feature predictor to a global risk stratification tool. These findings extend current understanding of the biological architecture of aggressive lung adenocarcinoma and reframe the clinical utility of preoperative metabolic imaging.

The most striking finding of our co-occurrence analysis was the strong positive association between LVI and OLNM (observed-to-expected ratio 5.85; Phi = 0.41; *p* < 0.001), indicating that these two features occurred together nearly six times more often than expected under independence. This finding supports a biological continuum between lymphovascular invasion and nodal metastasis: LVI represents histological evidence of tumor cells gaining access to the lymphatic or vascular system, a process anatomically linked to regional lymph node involvement. Consistently, Sugita et al. demonstrated that lymphatic permeation and vascular invasion predict distinct recurrence patterns: ipsilateral mediastinal nodal recurrence and distant organ metastasis, respectively [3]. This supports the interpretation that LVI-positive tumors frequently coexist with occult nodal disease. Consistently, a recent study showed that PET/CT-derived features can predict lymphovascular invasion in non-small cell lung cancer [19]. This association carries direct clinical implications. First, it provides a rationale for considering more extensive intraoperative lymph node dissection in patients with suspected LVI, even when preoperative imaging does not indicate nodal involvement, although this hypothesis awaits prospective validation. Second, it suggests that biomarkers predictive of LVI, such as the elevated SUVmax identified in our study, may serve as indirect indicators of occult nodal disease risk. This concept is corroborated by recent evidence. Metabolic parameters on preoperative FDG PET/CT independently predict LVI in NSCLC [20], and SUVmax contributes to nodal risk assessment in early-stage lung adenocarcinoma [21,22]. These findings provide a rationale for integrating metabolic imaging into risk-adapted treatment algorithms.

The association between STAS and OLNM was the weakest in our matrix, although it remained positive (Phi = 0.17, *p* < 0.001). This may reflect the broader definition of OLNM in our cohort, which encompassed both N1 and N2 stations, whereas previous studies linking STAS to occult nodal disease focused specifically on intrapulmonary N1 involvement [23]. The relatively weaker coupling suggests that LVI-mediated dissemination targets regional nodal basins irrespective of station, whereas STAS-related nodal spread, if present, may be confined to intrapulmonary routes.

No negative associations were observed. Even the weakest pairs, STAS with VPI (Phi = 0.13) and the solid with the micropapillary component (Phi = 0.15), remained positively associated after correction for multiple testing. Our data therefore argue against mutually exclusive invasive phenotypes. The six high-risk factors tend to accumulate within the same tumor, with LVI and OLNM at the center of the network. This is consistent with these features representing partially overlapping manifestations of a shared invasive program [12]. It also argues against treating airspace dissemination and pleurovascular invasion as alternative and mutually exclusive routes.

Our analysis of SUVmax revealed a hierarchy of predictive value across the six high-risk factors. In multivariable logistic regression, SUVmax independently predicted all six factors, with adjusted odds ratios from 1.141 (micropapillary component) to 1.283 (solid component); all *p* < 0.001. The weakest association was observed for the micropapillary component, which is consistent with the histological architecture of that subtype: its low cellular packing density and abundant extracellular mucin may attenuate FDG avidity. A recent cohort study in resected lung adenocarcinoma also reported only a partial correspondence between preoperative SUVmax and the predominant histological pattern [24].

The effect size was largest for solid component, consistent with the well-established link between high-grade histology and increased glucose metabolism [25]. Solid growth patterns are characterized by dense cellularity and reduced stromal volume. These histological features create a microenvironment favoring aerobic glycolysis even under normoxic conditions, a metabolic shift directly reflected in elevated GLUT-1 expression and increased FDG avidity [25,26]. The moderate predictive value for LVI and OLNM likely reflects the association between elevated baseline glycolytic activity and invasive tumor biology. Once LVI is established, occult nodal seeding may occur through passive mechanical transport of tumor emboli, a process for which the incremental metabolic requirement may be modest. This may explain why the OR for OLNM (1.188) was modestly lower than that for LVI (1.223): SUVmax captures the metabolic phenotype that enables invasion, whereas nodal metastasis represents a subsequent anatomical event.

The association with STAS was among the strongest observed (adjusted OR 1.225), suggesting that airspace dissemination, although histologically a local phenomenon, accompanies metabolically aggressive tumor biology. This is consistent with recent evidence that STAS can be predicted preoperatively and carries independent prognostic weight in clinical stage IA disease [27]. The single-voxel nature of SUVmax, however, cannot capture the spatial heterogeneity of STAS.

Notably, the current study did not evaluate survival outcomes, as the receipt and modality of postoperative adjuvant therapy in patients with high-risk pathological features constituted major confounding factors that would have precluded meaningful interpretation of prognostic associations. In our cohort, patients harboring multiple high-risk factors were more likely to receive adjuvant chemotherapy or targeted therapy, interventions that independently modify recurrence risk and survival. Disentangling the prognostic effect of high-risk factor co-occurrence from the therapeutic effect of adjuvant treatment would require a prospectively designed study with standardized treatment protocols. Accordingly, we restricted the present analysis to the predictive value of SUVmax for pathological features, reserving the evaluation of treatment-modified survival outcomes as a key objective of future investigations.

The most clinically actionable finding of our study concerns the predictive performance of SUVmax for high-risk burden rather than individual factors. The AUCs for single factors ranged from 0.702 (micropapillary component) to 0.796 (LVI). High-risk burden (≥3 concurrent factors) reached 0.838, which was higher than the value for any single factor in this cohort. This reframes the role of preoperative PET/CT in lung adenocarcinoma staging: rather than attempting to predict whether a specific pathological feature such as STAS or micropapillary component is present, clinicians should use SUVmax as a global risk stratification tool to identify patients likely to harbor multiple concurrent high-risk features. At the optimal cut-off of 3.0, sensitivity was 86.8% and the negative predictive value was 96.8%, so that a low SUVmax makes three or more concurrent high-risk factors unlikely. However, because this analysis did not assess survival or treatment outcomes, such a role remains a hypothesis requiring prospective validation rather than a treatment recommendation. This “high-burden” concept aligns with the IASLC grading philosophy, which aggregates high-grade patterns to determine overall tumor aggressiveness [9].

Although SUVmax was independently associated with the micropapillary component, this association was the weakest of the six factors (adjusted OR 1.141; AUC 0.702). The micropapillary component is widely recognized as one of the most aggressive histological patterns in lung adenocarcinoma, associated with early metastasis, pleural dissemination, and poor survival. In patients with small (≤2 cm) tumors, the presence of even a minor micropapillary component (≥5%) significantly elevates the risk of recurrence after limited resection [28]. Its discriminative performance was nevertheless the lowest of the six factors, consistent with a radiomics study in which SUVmax alone distinguished poorly between tumors with and without a micropapillary component [29]. Several mechanisms may explain this dissociation. First, micropapillary tumors exhibit a distinctive architecture characterized by reverse polarity, with apical surfaces oriented toward the stromal compartment rather than the alveolar lumen [30], a configuration that may reduce FDG avidity. Second, the diminutive tumor nests typical of micropapillary growth may fall below the spatial resolution of PET imaging. Partial volume effects may then render their metabolic signal indistinguishable from background activity [31]. Regardless of the underlying mechanism, our findings serve as an important caution: clinicians should not be falsely reassured by a low SUVmax in patients with radiological or clinical suspicion of micropapillary-predominant tumors. In such cases, preoperative biopsy or intraoperative frozen section assessment of histological pattern may be warranted to guide surgical strategy.

The STAS detection rate showed a borderline increase from 2020 to 2023 (5.5% to 44.2%; nominal trend *p* = 0.047, not significant after correction). The detection rates of the other five factors remained stable. This likely reflects evolving pathological awareness and diagnostic standardization rather than a true biological shift in tumor behavior. During this period, STAS gained increasing recognition as an independent prognostic factor, prompting pathologists to scrutinize surgical specimens more carefully for airspace tumor deposits. This growing recognition culminated in the IASLC Staging Project’s formal recommendation to incorporate STAS as a histological descriptor in the ninth edition of the TNM classification, based on data from over 4000 patients [32]. This diagnostic evolution underscores that temporal trends in STAS reporting must be interpreted as surveillance artifacts rather than true epidemiological shifts. Adjusting the burden model for year of surgery did not materially change discrimination (AUC 0.834 versus 0.838). All pairwise associations also remained positive within each study year. The co-occurrence structure is therefore not an artifact of temporal reporting changes. Conversely, the OLNM detection rate showed no significant change over the study period (8.2% to 5.8%, trend *p* = 0.42), suggesting that preoperative nodal staging and the pathological recognition of occult nodal disease were already established at our institution during these years [33]. These findings underscore the importance of considering temporal diagnostic drift when interpreting retrospective pathology data.

Cross-tumor comparisons contextualize our approach. Co-occurrence and mutual-exclusivity analyses have been applied to other malignancies almost exclusively at the molecular level. Examples include the adverse prognostic effect of concurrent driver-gene mutations in pancreatic adenocarcinoma and TP53–EGFR mutual exclusivity in glioblastoma [34,35]. To our knowledge, however, the systematic quantification of co-occurrence among pathological high-risk factors using Phi coefficients has not been reported in any tumor type. Although SUVmax predicts individual pathological features such as lymphovascular invasion in endometrial and gastric cancer [36,37], no study has directed SUVmax toward factor co-occurrence or high-risk burden. Our finding that SUVmax performs best when targeting three or more concurrent factors therefore extends these precedents from single-factor prediction to an integrated, burden-based readout.

Several limitations should be acknowledged. First, this single-center retrospective study is inherently susceptible to selection bias. Tumors negative for all six high-risk factors were retained, but patients without preoperative PET/CT or with incomplete pathological reporting were excluded. The cohort may therefore not represent all resected lung adenocarcinomas; the reported thresholds should not be extrapolated to screening. Bootstrap internal validation supported the discrimination for high-risk burden (AUC 0.838, 95% CI 0.807–0.865), although the cut-off was less stable; external multicenter validation remains necessary. Second, the temporal trend in STAS detection suggests evolving pathological diagnostic standards during the study period, which may limit generalizability to other institutions with different reporting practices. Third, OLNM was analyzed as a binary variable without stratification by nodal station (N1 vs. N2) due to limited sample size in subgroups; future studies should explore whether SUVmax differentially predicts specific OLNM subtypes. Fourth, we relied on institutional pathology reports for high-risk factor extraction, and inter-observer variability in the identification of these factors is well documented. For STAS, a multicenter prospective study reported substantial interobserver agreement among pathologists (κ = 0.756) but only moderate agreement between frozen section and final pathology (κ = 0.575) [38]. For the IASLC grading system, interobserver reproducibility was moderate (κ = 0.617) in the original validation study [9]. Although disagreements in our cohort were resolved by consensus discussion, the retrospective design precludes formal inter-observer reliability assessment, which should be addressed in prospective studies with predefined reading protocols. Fifth, our analysis did not incorporate radiomic features from CT or PET images, which have been shown to improve the prediction of high-risk pathological features beyond SUVmax alone [39]. Finally, the absence of survival data in the current analysis precludes direct evaluation of whether the identified co-occurrence patterns and SUVmax thresholds translate into differential prognostic outcomes. Overall, the present findings are confined to pathological risk prediction and should not be interpreted as evidence of prognostic or treatment benefit.

Taken together, these findings indicate that the six high-risk factors tend to co-occur rather than to segregate, and that the primary clinical value of preoperative PET/CT lies in identifying patients with high-risk burden (three or more concurrent factors) rather than in predicting individual pathological features.

Future work should pursue two directions. First, the co-occurrence structure characterized here, in particular the central position of LVI and OLNM, may define risk groups [40] that could guide adjuvant therapy decisions. Second, CT-derived features, volumetric PET parameters (SUVmean, MTV, TLG), and texture features could be integrated with SUVmax in multimodal machine learning models. Such models may add predictive value beyond any single modality, as recent radiomics studies support [39,41]. Together, these approaches may bridge the gap between postoperative histological assessment and preoperative clinical decision-making.

## 5. Conclusions

Lung adenocarcinoma exhibits widespread positive co-occurrence among pathological high-risk factors, with lymphovascular invasion and occult lymph node metastasis forming the center of the network, and no evidence of mutual exclusivity. Preoperative SUVmax is independently associated with all six high-risk factors. It performs best in identifying high-risk burden (three or more concurrent factors). At a cut-off of 3.0, the AUC was 0.838 and the negative predictive value was 96.8%. This risk-stratified approach requires validation in prospective cohorts with survival endpoints before it can inform preoperative decision-making in resectable lung adenocarcinoma. Whether this burden-based stratification can guide preoperative management requires prospective multicenter studies with survival endpoints. Integrating volumetric PET parameters and CT-derived radiomic features is the natural next step.

## Figures and Tables

**Table 1:** Baseline characteristics of 1292 patients with resected invasive lung adenocarcinoma, stratified by the number of concurrent high-risk factors.

Characteristic	0 Factors	1 Factor	2 Factors	3 Factors	≥4 Factors
n	635	260	207	114	76
Age (years), median (IQR)	60 (54–66)	60 (54–67)	60 (55–67)	59 (54–67)	60 (55–66)
Male, n (%)	222 (35.0%)	125 (48.1%)	105 (50.7%)	61 (53.5%)	31 (40.8%)
SUVmax, median (IQR)	1.3 (0.0–2.3)	2.3 (1.4–4.6)	4.5 (2.3–7.1)	6.8 (3.6–10.1)	7.3 (4.3–10.9)
Tumor size (mm), median (IQR)	18 (14–23)	20 (16–25)	21 (17–25)	23 (17–29)	24 (19–28)
STAS, n (%)	0 (0.0%)	84 (32.3%)	144 (69.6%)	91 (79.8%)	66 (86.8%)
VPI, n (%)	0 (0.0%)	51 (19.6%)	46 (22.2%)	50 (43.9%)	50 (65.8%)
LVI, n (%)	0 (0.0%)	2 (0.8%)	12 (5.8%)	22 (19.3%)	56 (73.7%)
OLNM, n (%)	0 (0.0%)	13 (5.0%)	17 (8.2%)	27 (23.7%)	51 (67.1%)
Solid component, n (%)	0 (0.0%)	57 (21.9%)	76 (36.7%)	66 (57.9%)	52 (68.4%)
Micropapillary component, n (%)	0 (0.0%)	53 (20.4%)	119 (57.5%)	86 (75.4%)	57 (75.0%)

**Table 2:** Prevalence of six pathological high-risk factors and distribution of concurrent factor burden (n = 1292). Confidence intervals were calculated using the Clopper–Pearson exact method.

Factor	Positive Cases	Total	Prevalence (%)	95% CI
STAS	385	1292	29.8	27.3–32.4
VPI	197	1292	15.2	13.3–17.3
LVI	92	1292	7.1	5.8–8.7
OLNM	108	1292	8.4	6.9–10.0
Solid component	251	1292	19.4	17.3–21.7
Micropapillary component	315	1292	24.4	22.1–26.8
0 factors	635	1292	49.1	46.4–51.9
1 factor	260	1292	20.1	18.0–22.4
2 factors	207	1292	16.0	14.1–18.1
3 factors	114	1292	8.8	7.3–10.5
4 factors	52	1292	4.0	3.0–5.2
5 factors	20	1292	1.5	0.9–2.4
6 factors	4	1292	0.3	0.1–0.8

**Table 3:** Pairwise co-occurrence patterns of pathological high-risk factors (n = 1292). All 15 pairs showed positive associations. *p* values are Bonferroni-corrected for the 15 pairwise comparisons (α = 0.0033). Rows are ordered by the observed-to-expected ratio.

Factor A	Factor B	Co-Occurrence (n)	O/E Ratio	Phi Coefficient	*p* Value
LVI	OLNM	45	5.85	0.406	<0.001
VPI	LVI	41	2.92	0.226	<0.001
VPI	OLNM	44	2.67	0.214	<0.001
LVI	Solid component	45	2.52	0.206	<0.001
STAS	LVI	67	2.44	0.260	<0.001
OLNM	Solid component	50	2.38	0.205	<0.001
LVI	Micropapillary component	53	2.36	0.214	<0.001
STAS	Micropapillary component	211	2.25	0.462	<0.001
OLNM	Micropapillary component	50	1.90	0.154	<0.001
STAS	OLNM	60	1.86	0.170	<0.001
VPI	Solid component	70	1.83	0.173	<0.001
STAS	Solid component	126	1.68	0.219	<0.001
VPI	Micropapillary component	77	1.60	0.145	<0.001
Solid component	Micropapillary component	95	1.55	0.154	<0.001
STAS	VPI	87	1.48	0.133	<0.001

**Table 4:** Univariate association between SUVmax and each high-risk factor (Mann–Whitney U test).

Factor	Positive (n)	SUVmax in Positive, Median (IQR)	Negative (n)	SUVmax in Negative, Median (IQR)	*p* Value
STAS	385	4.5 (2.2–8.1)	907	1.7 (0.9–3.2)	<0.001
VPI	197	5.3 (3.0–8.8)	1095	1.9 (1.0–4.0)	<0.001
LVI	92	6.5 (3.8–10.8)	1200	2.1 (1.1–4.5)	<0.001
OLNM	108	6.5 (3.7–10.1)	1184	2.0 (1.1–4.5)	<0.001
Solid component	251	5.9 (3.1–9.6)	1041	1.9 (1.0–3.7)	<0.001
Micropapillary component	315	4.2 (2.2–7.3)	977	1.9 (1.0–3.9)	<0.001

**Table 5:** Multivariable logistic regression of variables associated with each pathological high-risk factor. Values are odds ratios (95% confidence intervals). Each column represents a separate model, adjusted for age, sex, nodule size and nodule location. The last column shows the subgroup analysis using a ≥20% threshold to define the solid component. * *p* < 0.05; ** *p* < 0.01; *** *p* < 0.001. AIC, Akaike information criterion.

Variable	STAS	VPI	LVI	OLNM	Solid Component	Micropapillary Component	Solid Component (≥20%)
Intercept	0.294 ** (0.116–0.740)	0.026 *** (0.008–0.087)	0.023 *** (0.004–0.121)	0.070 *** (0.016–0.307)	0.082 *** (0.026–0.256)	0.141 *** (0.054–0.368)	6.742 (0.778–58.427)
SUVmax	1.225 *** (1.178–1.274)	1.178 *** (1.131–1.226)	1.223 *** (1.164–1.286)	1.188 *** (1.134–1.245)	1.283 *** (1.229–1.339)	1.141 *** (1.101–1.183)	1.272 *** (1.172–1.381)
Age (years)	0.991 (0.976–1.006)	1.009 (0.990–1.028)	1.007 (0.981–1.033)	0.983 (0.960–1.007)	0.999 (0.981–1.017)	1.005 (0.990–1.020)	0.975 (0.943–1.007)
Male sex	1.403 * (1.081–1.820)	1.012 (0.729–1.404)	0.778 (0.490–1.236)	0.824 (0.537–1.263)	1.994 *** (1.464–2.716)	1.139 (0.872–1.487)	1.430 (0.799–2.559)
Nodule size (mm)	1.007 (0.987–1.027)	1.034 ** (1.009–1.059)	1.001 (0.968–1.036)	1.028 (0.997–1.060)	0.989 (0.966–1.013)	1.005 (0.984–1.025)	0.933 ** (0.893–0.975)
Upper lobe location	0.782 (0.600–1.018)	0.937 (0.671–1.307)	0.796 (0.502–1.260)	0.874 (0.570–1.339)	0.945 (0.688–1.298)	0.808 (0.617–1.057)	0.491 * (0.269–0.894)
AIC	1409.4	996.4	592.0	670.5	1069.0	1365.7	300.0
Pseudo-R^2^	0.107	0.104	0.123	0.111	0.166	0.052	0.152

**Table 6:** Discriminative performance of SUVmax for predicting high-risk factors and high-risk burden. Optimal cut-offs were determined by the Youden index.

Outcome	Analysis Type	AUC	Cut-Off	Sensitivity (%)	Specificity (%)	Positive Cases (n)	Total (n)
STAS	Primary analysis	0.746	2.7	70.9	69.1	385	1292
VPI	Primary analysis	0.763	2.8	77.7	64.3	197	1292
LVI	Primary analysis	0.796	2.7	88.0	60.7	92	1292
OLNM	Primary analysis	0.793	2.9	85.2	62.9	108	1292
Solid component	Primary analysis	0.780	2.9	77.3	67.6	251	1292
Micropapillary component	Primary analysis	0.702	2.8	67.3	66.0	315	1292
High-risk burden (≥3 factors)	Primary analysis	0.838	3.0	86.8	67.8	190	1292
Solid component (≥20%)	Sensitivity analysis	0.699	6.7	58.3	76.3	132	246

## Data Availability

The datasets generated and analyzed during the current study are available from the corresponding author upon reasonable request and with institutional approval, owing to privacy and ethical restrictions.

## Supplementary Materials

The following supporting information can be downloaded at: https://www.mdpi.com/article/10.3390/cancers18213435/s1, Table S1: Association between nodule location and pathological high-risk factors; Table S2: Association between nodule size and pathological high-risk factors; Table S3: Bootstrap internal validation of SUVmax cut-offs and diagnostic performance; Table S4: Year-stratified Phi coefficients for pairwise associations among pathological high-risk factors.

## Abbreviations

The following abbreviations are used in this manuscript:

AUC	area under the receiver operating characteristic curve
CI	confidence interval
EBUS-TBNA	endobronchial ultrasound-guided transbronchial needle aspiration
FDG	fluorodeoxyglucose
GLUT-1	glucose transporter 1
IASLC	International Association for the Study of Lung Cancer
IQR	interquartile range
LVI	lymphovascular invasion
MTV	metabolic tumor volume
NCCN	National Comprehensive Cancer Network
NSCLC	non-small cell lung cancer
O/E	observed-to-expected
OLNM	occult lymph node metastasis
OR	odds ratio
PET/CT	positron emission tomography/computed tomography
ROC	receiver operating characteristic
STAS	spread through air spaces
SUVmax	maximum standardized uptake value
TLG	total lesion glycolysis
VPI	visceral pleural invasion
WHO	World Health Organization
