# Supplementary material for: Co-Occurrence Patterns of Pathological High-Risk Factors and Predictive Value of PET Metabolic Features in Lung Adenocarcinoma: A Retrospective Cohort Study of 1292 Cases
Source: Cancers (Basel). 2026 Oct 23;18(21):3435. doi: 10.3390/cancers18213435 (PMC13761731; doi:10.3390/cancers18213435)
Supplement: Supplementary file 1 [file cancers-18-03435-s001.zip › cancers-4627974-supplementary.pdf]

Supplementary Materials

Table S1. Association between nodule location and pathological high-risk factors.

Factor	Upper lobe (n)	Upper lobe (%)	Non-upper lobe (n)	Non-upper lobe (%)	P value	Significance
STAS	779	28.8	513	31.4	0.3204	nan
VPI	779	15.5	513	14.8	0.7522	nan
LVI	779	6.8	513	7.6	0.5825	nan
OLNM	779	8.2	513	8.6	0.8376	nan
Solid component	779	20.0	513	18.5	0.5185	nan
Micropapillary component	779	23.4	513	25.9	0.3206	nan

Table S2. Association between nodule size and pathological high-risk factors.

Factor	≥22 mm (n)	≥22 mm (%)	<22 mm (n)	<22 mm (%)	P value	Significance
STAS	518	37.3	774	24.8	<0.001	***
VPI	518	21.8	774	10.9	<0.001	***
LVI	518	10.6	774	4.8	<0.001	***
OLNM	518	12.2	774	5.8	<0.001	***
Solid component	518	26.1	774	15.0	<0.001	***
Micropapillary component	518	29.9	774	20.7	<0.001	***

Table S3. Bootstrap internal validation of SUVmax cut-offs and diagnostic performance.

Outcome	n	Positive (n)	AUC	AUC 95% CI	Cut-off	Cut-off 95% CI	Sensitivity (apparent)	Sensitivity (corrected)	Specificity (apparent)	Specificity (corrected)
STAS	1292	385	0.746	0.717-0.773	2.7	2.60-3.40	0.709	0.703	0.691	0.689
VPI	1292	197	0.763	0.727-0.797	2.8	2.00-4.00	0.777	0.759	0.643	0.640
LVI	1292	92	0.7	0.752-	2.	2.50-	0.880	0.859	0.607	0.605

			96	0.833	7	4.10				
OLNM	1292	108	0.793	0.757-0.831	2.9	2.80-4.10	0.852	0.835	0.629	0.627
Solid component	1292	251	0.780	0.747-0.809	2.9	2.70-4.50	0.773	0.761	0.676	0.674
Micropapillary component	1292	315	0.702	0.667-0.734	2.8	2.10-3.20	0.673	0.663	0.660	0.657
High-risk burden (≥3 factors)	1292	190	0.838	0.807-0.865	3.0	2.70-4.10	0.868	0.856	0.678	0.676

Table S4. Year-stratified Phi coefficients for pairwise associations among pathological high-risk factors.

Pair	Overall	2020	2021	2022	2023
STAS × VPI	+0.133	+0.008	+0.060	+0.170	+0.294
STAS × LVI	+0.260	+0.332	+0.268	+0.248	+0.265
STAS × OLNM	+0.170	+0.293	+0.245	+0.169	+0.161
STAS × Solid component	+0.219	+0.101	+0.096	+0.259	+0.244
STAS × Micropapillary component	+0.462	+0.152	+0.279	+0.473	+0.499
VPI × LVI	+0.226	+0.269	+0.081	+0.355	+0.264
VPI × OLNM	+0.214	+0.148	+0.222	+0.298	+0.164
VPI × Solid component	+0.173	+0.146	+0.295	+0.129	+0.107
VPI × Micropapillary component	+0.145	+0.166	+0.110	+0.188	+0.194
LVI × OLNM	+0.406	+0.333	+0.311	+0.440	+0.557
LVI × Solid	+0.206	+0.211	+0.150	+0.202	+0.264

component					
LVI × Micropapillary component	+0.214	+0.285	+0.236	+0.252	+0.144
OLNM × Solid component	+0.205	+0.194	+0.261	+0.169	+0.188
OLNM × Micropapillary component	+0.154	+0.022	+0.210	+0.206	+0.129
Solid component × Micropapillary component	+0.154	+0.134	+0.041	+0.194	+0.143
